# Of Oestrogens and Sperm: A Review of the Roles of Oestrogens and Oestrogen Receptors in Male Reproduction

**DOI:** 10.3390/ijms18050904

**Published:** 2017-04-25

**Authors:** Pavla Dostalova, Eva Zatecka, Katerina Dvorakova-Hortova

**Affiliations:** 1 Group of Reproductive Biology, Institute of Biotechnology CAS, v.v.i., BIOCEV, Prumyslova 595, 25250 Vestec, Czech Republic; pavla.dostalova@ibt.cas.cz (P.D.); eva.zatecka@ibt.cas.cz (E.Z.); 2 Department of Zoology, Faculty of Science, Charles University, Vinicna 7, 12844 Prague 2, Czech Republic

**Keywords:** oestrogens, oestrogen receptors, testes, sperm, signalling, oestrogen-like compounds, humans, mice, rats, pigs

## Abstract

The crucial role that oestrogens play in male reproduction has been generally accepted; however, the exact mechanism of their action is not entirely clear and there is still much more to be clarified. The oestrogen response is mediated through oestrogen receptors, as well as classical oestrogen receptors’ variants, and their specific co-expression plays a critical role. The importance of oestrogen signalling in male fertility is indicated by the adverse effects of selected oestrogen-like compounds, and their interaction with oestrogen receptors was proven to cause pathologies. The aims of this review are to summarise the current knowledge on oestrogen signalling during spermatogenesis and sperm maturation and discuss the available information on oestrogen receptors and their splice variants. An overview is given of species-specific differences including in humans, along with a detailed summary of the methodology outcome, including all the genetically manipulated models available to date. This review provides coherent information on the recently discovered mechanisms of oestrogens’ and oestrogen receptors’ effects and action in both testicular somatic and germ cells, as well as in mature sperm, available for mammals, including humans.

## 1. Introduction

Oestrogens are steroid hormones that exhibit pleiotropic effects, among which their best-known role is to control the oestrous/menstrual cycle of females. Therefore, oestrogens are often referred to as female sex hormones. Nevertheless, oestrogens are also present in males and the main source of their endogenous production lies in the testes. This male reproductive organ synthesises an enzyme called aromatase, which is responsible for the irreversible conversion of testosterone into oestrogens. Due to this fact, relatively high levels of oestrogens are present at the site of sperm production, and they are even higher than serum levels in females [1]. For general interest, the concentration of 17β-estradiol (E2) is 2–25 pg/mL in the blood plasma of male rats, but it is 250 pg/mL in the rete testis fluid [2,3]. The serum level of E2 in female rats varies between 30 and 90 pg/mL depending on the phase of the oestrous cycle [4]. Similarly, in men, peripheral vein E2 concentration is around 20 pg/mL, but 50 times higher (1 ng/mL) in the spermatic vein [5]. On the other hand, in non-pregnant pre-menopausal women the plasma E2 concentration ranges from 50 to 400 pg/mL, depending on the menstrual cycle [6].

For a long time, oestrogens were not considered important in male reproduction, until it was shown that a gene disruption of the oestrogen receptor leads to decreased fertility in male mice [7]. Since this turning point, considerably more attention has been given to the topic and there have been many studies dedicated to oestrogen signalling in males. At present, we know that E2 binds either to classical intracellular oestrogen receptors (ERs) or to a membrane oestrogen receptor such as G protein-coupled oestrogen receptor 1 (GPER, GPR30), and therefore it can trigger both the genomic and non-genomic signalling pathways. These pathways may be disrupted by several factors including environmental pollutants. Many of these compounds have the ability to mimic endogenous oestrogen behaviour and thus can alter physiological oestrogen signalling. According to the World Health Organisation (WHO) standards, the male factor plays a crucial part in decreasing human fertility [8]. At the same time, there is growing evidence that some environmental pollutants are able to influence the male reproductive system (see review [9,10]) and even reprogram the sperm epigenome [10,11,12,13]. Therefore, a potential connection between globally increasing environmental pollution and decreasing semen quality could be made. For this reason, it is important to know the exact mechanism of oestrogen signalling and its function in male reproduction to fully comprehend the external stimuli that can modify its action. Moreover, this knowledge can help us to further understand the mechanism of sperm production and perhaps even prevent the negative effects caused by environmental pollutants.

Oestrogen receptors are crucial for oestrogen signalling and it has been shown that they are expressed in both somatic and germ testicular cells, as well as in mature sperm. In addition to wild-type ERs, several variants/isoforms of ERs with distinct DNA- or ligand-binding properties have been described [14,15,16,17,18,19,20,21,22,23,24,25,26,27,28,29]. These variants/isoforms originate from alternative splicing or alternative use of promoters, 3′-coding and 5′-non-coding regions [30]. Therefore, the knowledge of functional variants that can specifically modulate the oestrogen response is crucial for comprehending oestrogen action. Thus, this review aims to summarise the current knowledge about oestrogen signalling in the male reproductive tract, with a special focus on oestrogen receptors and their function, as well as briefly discuss the most potent oestrogen like-compounds with proven adverse interactions with oestrogen receptors.

## 2. Oestrogen Receptors

Oestrogen signalling is a complex process that depends on the cell milieu and presence of receptors. Currently, two types of classical oestrogen receptors, namely oestrogen receptor 1/alpha (ESR1, ERα) and oestrogen receptor 2/beta (ESR2, ERβ), as well as the transmembrane receptor GPER, are known.

Classical ERs belong to group A of nuclear receptor subfamily 3 and their protein is composed of five domains: N-terminal A/B domain with transactivation function one (AF-1), DNA-binding domain (DBD), hinge domain, ligand-binding domain (LBD) with transactivation function two (AF-2), and C-terminal domain. ERα and ERβ are products of different genes, but they share considerable homology and bind E2 with a similar affinity (see review [31,32]). On the other hand, some differences in the LBD of ERs result in a different affinity to various estrogenic compounds (see review [33]). In addition to wild-type variants, several splice variants with different DNA- and ligand-binding properties have also been described [14,15,16,17,18,19,20,21,22,23,24,25,26,27,28,29].

GPER is a 7-transmembrane protein that belongs to the G protein-coupled receptor (GPCR) superfamily and it is not only located in the plasma membrane [34,35], but it has also been detected in the membranous organelles such as endoplasmic reticulum [36] and Golgi apparatus [37]. GPER mediates oestrogen-dependent cellular activation and was found to be expressed in several tissues [38,39,40,41], including those of the male reproductive tract [42,43,44]. Activation of GPER triggers so-called rapid non-genomic signalling pathways, and cellular crosstalk between GPER receptor and classical ERs mediated by oestrogens has been suggested [45,46]. Interestingly, their interactions seem to depend on the cellular context and also on the oestrogen concentration (see review [47]).

It is relevant to mention that except for the well-known classical ERs and GPER, several novel membrane-associated receptors such as ER-X [48], ER-x [49] and STX-binding protein [50] have been proposed as oestrogen receptors. Since the definition of ERs is missing, it remains a matter of debate whether these later described receptors are really oestrogen receptors [51], and their further characterization is required. ER-X has a sequence homology to ERα, binds estradiol with high affinity, activates extracellular signal-regulated kinases ERK1/2, is developmentally regulated in the neocortex and uterus with maximal expression at postnatal days 7–10, and may be re-expressed in the adult brain after injury [48]. ER-x was described as a membrane ER that mediates oestrogen signalling in some breast cancer cell lines, leading to the modulation of apoptosis, growth factor signalling and transcriptional regulation [49]. Whether these receptors also play a role in male reproduction remains to be determined.

### 2.1. Signalling Pathways

**Figure 1:**
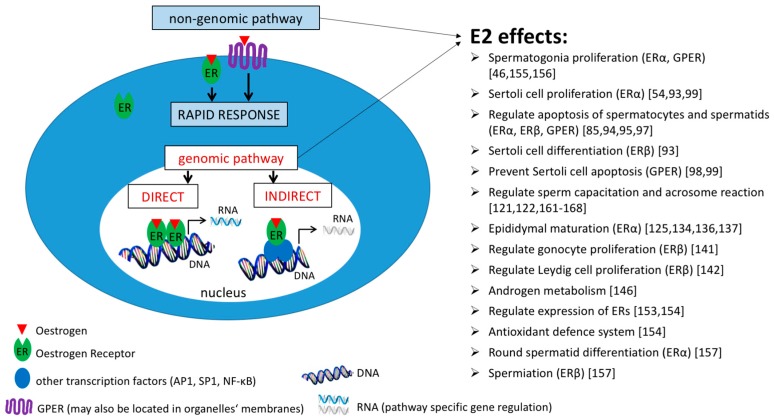
Oestrogen signalling pathways. Genomic pathway (white boxes): E2–ER binding leads to the activation of the relevant receptors, formation of homo- or hetero-dimers, binding to ERE (oestrogen response element) in the promotor sequence of a target gene and regulation of its transcription. Alternatively, E2-ER can bind to other transcription factors such as AP-1, Sp-1 or NF-κB, through which they indirectly regulate the expression of the target gene. Non-genomic pathway (blue boxes): E2 binds to receptors localised in the membrane (GPER) or its proximity (ERα, ERβ), which results in rapid cellular responses such as activation of protein kinases, regulation of ion channels or production of second messengers. Both pathways play an important role in oestrogen-mediated events in the testis, and the non-genomic one also in sperm; the known E2 effects are listed on the right-hand side of the diagram.

Oestrogens, due to their steroid nature, can pass through the plasma membrane and bind to intracellular receptors, which activate and trigger cellular responses. Within cells, including male germ cells, there are several pools of classical oestrogen receptors, generally clustered as nuclear, membrane and/or membrane-associated and cytosolic, as well as others, so-called non-classical/oestrogen related receptors. After oestrogen stimuli, ERs may relocalise within the nucleus and bind to DNA, as in the case of the genomic pathway [45,52,53], or ERs translocate to the membrane, where they may mediate non-genomic signalling [54]. E2 signalling pathways and a list of E2 effects are briefly summarised in Figure 1.

#### 2.1.1. Genomic Pathway

Binding of oestrogens to the classical nuclear oestrogen receptor results in a change of receptor conformation and its dissociation from heat shock proteins. Dissociated receptors form dimers and bind to the DNA sequence found in the regulatory region of oestrogen-responsive genes known as “oestrogen response element” (ERE). If both ERs are co-expressed within one cell, ERs may form homo- (αα, ββ) as well as hetero- (αβ) dimers [55]. Things get more complicated with the addition of the wild-type forms of classical ERs and several isoforms originating from alternative splicing that have been described in oestrogen-responsive tissues or cells [14,15,16,17,18,19,20,21,22,23,24,25,26,27,28,29]. Since ERs can form dimers, some ER variants may show a modulating function. For example, mouse variant ERβ2 has a 30-fold lower affinity to E2 than ERβ1, whereas ERβ2 is capable of forming dimers with ERα or ERβ1 and thus inhibits its transcriptional activity [22]. Similarly, a human ERβ2 variant (also known as hERβcx) that results from alternative splicing of exon 8 and has no ligand-binding ability shows dominant negative activity against ERα transactivation [24]. The question remains whether both ERβ variants, as well as ERα, are co-expressed within one type of testicular cell, and therefore can potentially interact, or whether their expression pattern varies depending on the germ cell differentiation stage. In addition, ERs may also regulate gene expression via binding to other transcription factors such as nuclear transcription factor-κB (NF-κB), stimulating protein-1 (Sp-1), and activator protein 1 (AP-1) (see review [53]).

#### 2.1.2. Non-Genomic Pathway

In addition to the genomic pathway, a non-genomic oestrogen pathway has been discovered in various somatic cells (see review [45,53,56,57]). In comparison with the genomic pathway, non-genomic signalling occurs rapidly within seconds to minutes and involves production of second messengers, activation of protein kinases, and modulation of ion-channels (see review [56,58]). Unlike the genomic pathway, non-genomic action assumes membrane association and/or localization of receptors. Compared to GPER, the classical ERs do not contain a hydrophobic part that may serve as a transmembrane domain [57]. Despite this fact, several groups have reported the presence of full-length ERs and also their variants in the membrane of somatic and cancer cells [59,60,61,62,63,64,65,66]. It has been demonstrated that these membrane receptors are the same proteins as the nuclear ones [61,62,63,64,66,67]. The translocation of ERs to the plasma membrane is mediated by the SRC family of tyrosine kinases [54]. Moreover, several groups have identified specific motifs [65,68] and modifications (palmitoylation [59,69,70], myristoylation [71]) that are responsible for the membrane localization of ERs. It has been hypothesised that membrane ERα, representing 5–10% of the entire endogenous pool of ERα, mediates the rapid oestrogen signalling [64]; however, in most cases, this signalling is attributed to GPER [72]. The knowledge on how the classical ERs translocate to the membrane together with the knowledge on GPER action might be of great importance for understanding this membrane-originated non-genomic oestrogen signalling.

### 2.2. Oestrogen Receptors in the Testis

**Figure 2:**
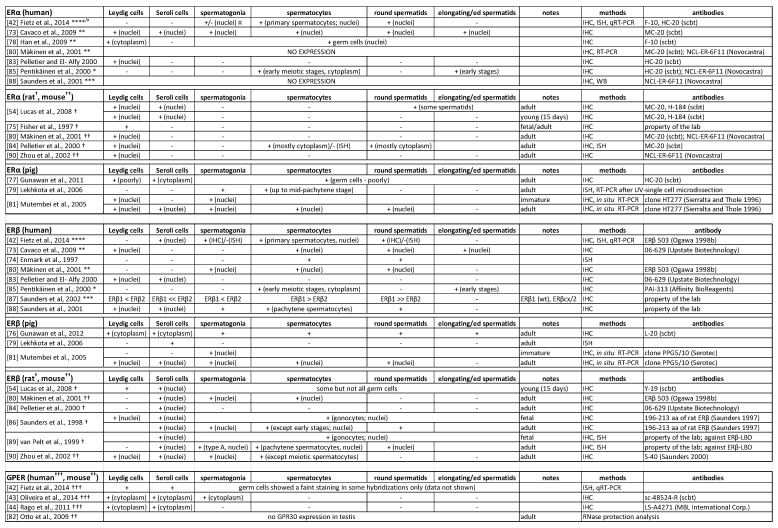
Oestrogen receptor localization in the testis. The Figure summarises up-to-date information on the localization of ERα, ERβ and GPER in human, pig, rat and mouse testicular cells. * Elderly men (60–80 years old) undergoing surgery due to prostatic cancer; ** azoospermia, maturation arrest, Sertoli cell-only; *** azoospermia, vasectomy; **** azoospermia, patients undergoing vasectomy; ¤ positive or negative ERα detection in spermatogonia depending on the approach used: IHC—positive staining with HC-20 antibody, negative staining with F-10 antibody, ISH—positive; † rat; †† mouse; ††† human; IHC—Immuno Histo Chemistry; ISH—In Situ Hybridization; qRT-PCR—quantitative Reverse Transcription Polymerase Chain Reaction; WB—Western Blot detection; scbt—Santa Cruz Biotechnology.

In the current literature, we can find evidence of the presence of ERs in all testicular cell types [42,43,44,54,73,74,75,76,77,78,79,80,81,82,83,84,85,86,87,88,89,90], although the published results often differ between species and studies (Figure 2). This variability of results in the expression of ERs can be caused by the presence of ER variants, other proteins that share homology with classical ER, or by used methodologies and antibodies. In addition, not all commercially available antibodies are exclusively specific, as evident from the study of multiple ERβ antisera that stained mouse tissues in ERβ knock-out animals (ERβKO) [91]. Therefore, one needs to be careful in selecting the appropriate antibodies, and parallel sequencing of the detected proteins is necessary.

The expression of aromatase, ERα and ERβ depends on the age, cell type, and apart from ERβ, on the stage of seminiferous epithelium [92]. Both ERs are expressed more extensively in adult testes compared to the juvenile ones. However, this expression pattern is not the same for all testicular cell types. In the case of Sertoli cells, the expression level of ERα declines, while the expression of ERβ increases with age [92,93]. This switch between ERα and ERβ expression allows E2 to mediate its effects in distinct directions and regulate proliferation of immature Sertoli cells, while later the same signal pushes these cells towards differentiation [93]. Interesting information comes from the study of infertile men suffering from azoospermia [73]. Biopsy of their testicular tissue confirmed the presence of both ERα and ERβ in somatic and germ cell types, supporting the conservative nature of ERs to control the testicular function, which has not been compromised by pathological spermatogenesis.

It is of interest that GPER was recently detected in the cytoplasm of rat pachytene spermatocytes [94], round spermatids [95] and Sertoli cells [96], as well as mouse spermatocyte-derived cell line, GC-2 [97], and spermatogonial-derived cell line, GC-1 [46]. It was also proven to be expressed in human Leydig and Sertoli cells [42,44], specifically in the endoplasmic reticulum and Golgi apparatus of Sertoli cells [96]. Several in vitro studies showed the involvement of GPER in the regulation of spermatogonia proliferation [46] and apoptosis of spermatocytes, spermatids [94,95,97] and Sertoli cells [98,99]; thus GPER may represent another receptor for oestrogen signalling in the male gonads. However, GPER knockout male mice are normally fertile [82,100], which may indicate a minor or substitutable role of GPER in the testicular physiology. GPER expression in the male reproductive tract needs further investigation to determine whether GPER expression, similarly to ERα and ERβ, depends on the age and the stage of seminiferous epithelium.

Sensitivity to oestrogen hormones also depends on availability of oestrogen receptor co-regulators. E2 treatment leads to an increase in the recruitment of ERβ and its co-repressor NCoR1 (nuclear receptor co-repressor 1) to the ERE of Arpc1b (actin related protein 2/3 complex subunit 1B) gene, thus causing downregulation of Arpc1b transcription in testes. In contrast, the recruitment of ERβ and its co-activator Src1 (steroid receptor co-activator 1) is decreased in Evl (Ena-vasodilator stimulated phosphoprotein) ERE after E2 treatment, thus causing downregulation of Evl [101]. Both, Arpc1b and Evl are involved in actin remodelling during spermiation, and they are responsive to E2 but not to dihydrotestosterone [102]. Src1, Src2 (also named TIF2, GRIP1), and Src3 (also named p/CIP, RAC3, ACTR, AIB1) belong to the p160 family of nuclear receptor co-activators. Even though the Src1 null male mouse mutants are fertile, they have a smaller but histologically normal testis [103]. The effect is more pronounced in the TIF2^−/−^ (Src2^−/−^) male mice, which have impaired fertility with defects in spermiogenesis and age-dependent testicular degeneration [104]. It is appropriate to mention that oestrogen receptor co-regulators may also be recruited by other nuclear receptors [105], and thus the effect of inactivation of these co-regulators may not only reflect the role of E2 signalling, but rather it may be more widespread.

Fatty acid amide hydrolase (Faah) controls the level of endocannabinoids [106], which affect the male reproductive function [107,108,109,110], and it has been identified as a direct target of E2 signalling in the testis [111]. The E2-induced increase of the Faah expression in Sertoli cells is a result of epigenetic modifications in the Faah promotor. Furthermore, E2 induces ERβ binding and histone demethylase LSD1 (histone lysine demethylase 1) recruitment at ERE2/3 sites in the Faah proximal promotor [111]. These findings suggest that a recruitment of the epigenetic modifiers is also involved in the E2 regulation of gene expression.

### 2.3. Oestrogen Receptors in Sperm

Several groups have tried to detect ERs in sperm to clarify the role of oestrogens on sperm; however, the results are not always in agreement. The first study confirming the binding of steroid hormones to sperm membranes date back to 1979 [112]. The binding sites were located on the midpiece, less on the head and neck, and least on the principal- and end-piece of the tail [113]. In the same year as the steroid hormone binding sites were described, the presence of cytosolic or nuclear ERs in human sperm was excluded [114]. ERs in human spermatozoa were for the first time detected nearly two decades later [115]. Nevertheless, results from the detection of ERs vary both between species and between studies, where differences can be found in molecular weight and localization.

By using different antibodies against ERα, a full-length protein of 66 kDa [115,116], or its truncated variant of 46 kDa [20], or both forms [117] were detected in human mature sperm. Interestingly, ERα detection in human germ cells (that were obtained from semen samples with more than 20% of round cells and were deprived of sperm) showed both forms, 46 and 66 kDa, to be present [20]. The expected molecular weight of human ERα protein is 66 kDa. The 46 kDa band may thus represent a splice form of ERα [118]. The human ERα protein has been localised in the tail [115], mid-piece only [116,119], and equatorial segment and tail [117]. In pig sperm, ERα was immunolocalised in the tail (predominantly in the midpiece) and it was detected as a 67 kDa band [120].

Concerning ERβ, according to the Uniprot database, the calculated molecular weight of ERβ isoform 1 is 59 kDa for the human, boar and mouse. In the case of human sperm, ERβ has been detected in the sperm tail [116,117,119], representing a band of 59 kDa [116], 64 kDa [117], and 55 kDa [119]. However, Lambard et al. [20], managed to detect two bands with an antibody against ERβ (50 and 60 kDa) only in round spermatids and not in mature human spermatozoa. In parallel to human, ERβ was also found in boar (50 and 59 kDa) [120] and mouse (64 kDa) [121] sperm, but compared to human, the immunolocalization was confined exclusively to the acrosomal region [120,121]. Certain differences in the detected molecular weight of ERβ could be caused by either detection of specific novel splice variants, which unfortunately have not been described in more detail, or simply by using a different methodology or antibodies of different origin (See Figure 2), which might have cross-labelled other proteins. It is highly probable that a yet uncharacterised protein sharing homology with ERβ exists and cross-reacts with many ERβ antibodies [91]. Thus, one must be careful when detecting the ERβ protein, and the tissues from knockout animals should be included in these studies as they represent the best negative control for work with ERβ antibodies.

A protein of much smaller size (29 kDa) was identified on the human sperm membrane using an antibody against the ligand-binding domain of a genomic oestrogen receptor. This protein was described as a novel oestrogen receptor that may mediate oestrogen effects in the sperm [122]; however, no further studies have investigated this in more detail.

### 2.4. Genetically Manipulated Mouse Models

A conclusive proof of oestrogen importance in male reproduction was delivered by knockout (KO) mice, where either production of oestrogens (aromatase knockout—ArKO) or oestrogen responsiveness (ERαKO, ERβKO, and ERαβKO) was disrupted. These genetically modified males were infertile/sterile [123,124,125], although some ERβKO remained fertile [124,126].

#### 2.4.1. Aromatase Knockout

As discussed previously, oestrogens can act via binding to various oestrogen receptors, and moreover, this binding may trigger different signalling pathways depending on the cellular content [127]. ArKO seems to be the best model for studying the role of oestrogens in male reproduction as there is no production of oestrogens in these individuals. ArKO male mice are initially fertile [128] but develop infertility due to progressively disrupted spermatogenesis between 18 weeks and one year of age, despite no decrease of gonadotrophins or androgens [129]. Spermatogenesis was reported to be disrupted mainly at the spermatid stage, while there was no observed effect in Sertoli cells and early germ cells, which indicates that oestrogens are required in the later stages of spermatogenesis. This has been supported by the discovery of ERs playing an important role in spermatid differentiation and spermiation [130]. Furthermore, ArKO males are reported to have an impaired sexual behaviour, which contributes to decreased fertility in the ArKO male mice [131,132].

#### 2.4.2. ERα Gene-Manipulated Models

Knock-out mice lacking ERs have become additional models for studying the role of oestrogens. Nevertheless, one should keep in mind that ERs can be activated even in the absence of oestrogens [133]; therefore, these models mainly provide knowledge about the importance of ERs themselves. There is no doubt that the ERα protein is necessary for male reproduction as ERαKO male mice are infertile [125]. Infertility is a consequence of damaged fluid reabsorption in the rete testis and the proximal part of the epididymis, which results in fluid accumulation in the lumen and back pressure in the testes, ultimately leading to testicular atrophy [125,134]. The degeneration of seminiferous tubules is progressive, starting at 10–12 weeks of age [125]. The question remains whether the ERα protein is also required during the process of spermatogenesis or whether its main role lies in fluid reabsorption. It was demonstrated that ERαKO germ cells are able to develop normally after transplantation to the testis of wild-type males depleted of germ cells [135]. Thus, it has been proven that ERα is not required by germ cells themselves, but the expression by somatic cells is crucial. However, it is not clear for which type of testicular and/or epididymal somatic cells ERα is indispensable. Furthermore, ERαKO mice have a reduced sperm count and motility and they are unable to fertilise eggs in vitro [125]; in addition, these sperm also exhibit abnormal flagellar coiling and increased incidence of spontaneous acrosome reaction [136]. The reduced sperm motility and abnormal flagellar coiling were shown to be the results of sperm exposure to an abnormal epididymal environment of hypo-osmolality and high pH [136,137], which indicates that certain functional defects of ERαKO sperm may have an epididymal origin. Recently, most abnormalities observed in ERαKO mice were also found in nuclear-only oestrogen receptor 1 (NOER) mice, although the phenotype was less severe. This indicates the importance of the membrane, as well as the nuclear ERα in male reproduction [138].

Another useful tool for the study of ERα is an oestrogen nonresponsive ERα knock-in (ENERKI) model, where the interaction of endogenous oestrogens with ER is significantly reduced [139]. Using ENERKI, it has been demonstrated that ligand-independent ERα signalling pathways are crucial for fluid reabsorption, explaining the absence of defects in fluid reabsorption in ArKO males. On the other hand, oestrogen-mediated ERα signalling is essential for germ cell viability and maintenance of the normal organization of seminiferous epithelium, most likely via support of the Sertoli cell function [139]. These findings emphasise the importance of oestrogen-dependent and independent mechanism of ERα action in the male reproductive tract. Moreover, it has been shown that also neonatal E2/ERα signalling is crucial for adult male fertility [139].

#### 2.4.3. ERβ Knockout

Despite the fact that ERβ is a predominant variant of ERs in the testis [140], knock out studies revealed that most of the generated ERβKO male mouse models are fertile. However, unfortunately, most analyses were performed in males on the prostate only, or focused exclusively on females. Therefore, it is possible that the role of ERβ in the testis biology has yet not been comprehensively investigated. First produced ERβKO (ERβKO_CH, Chapel Hill laboratory, University of North Carolina, Chapel Hill, NC, USA_) mice carried the neomycin-resistance gene in exon 3, and thus the first zinc-finger of DNA-binding domain was disrupted [126]. ERβKO_CH_ males are normally fertile, as assessed by the presence of a seminal plug, number and size of the litter. Nevertheless, a male fertility test was carried out between the ages of 6 and 12 weeks [126], which was the age when ArKO males were also classified as fertile, although later they developed progressive infertility [129]. Krege et al. [126] observed an interesting phenomenon related to splice variants in the ERβKO_CH_ mice. Although no alternative splicing was observed in the prostate, several splice variants were detected in the ovaries. Therefore, it is possible that splice variants could also exist in the testis and may compensate for the lack of full-length ERβ.

ERβKO (ERβKO_ST, Strasbourg laboratory, Strasbourg, France_) also targeted exon 3 [124]. Consistent with the previous study [126], ERβKO_ST_ males were fertile between the age of 7–15 weeks and the testicular histology was normal [124]. This was probably the reason why the study was mainly focused on females. Interestingly, only the full-length ERβ was expressed in the ovaries of wild-type mice, while three splice variants were present in the ERβKO ones. ERβKO_ST_ mice were also used to focus on the role of ERβ during foetal/neonatal life [141]. Similarly to the adult mice of ERβKO_ST_ [124] and ERβKO_CH_ [142], the testicular histology of neonates ERβKO_ST_ appeared to be phenotypically normal [141]. However, neonates’ heterozygous and homozygous ERβ gene inactivation increased the number of gonocytes, while no changes in the number of Leydig or Sertoli cells were observed [141]. On the other hand, adult ERβKO_CH_ males showed an increased Leydig cell count with a concomitant decrease of the mean cell volume, which probably explains why there were no changes in testosterone production. Furthermore, inactivation of ERβ led to an increased number of spermatogonia in adults, while there were no changes in the number of spermatocytes or spermatids [142]. The higher number of spermatogonia was probably due to the failure of ERβ signalling during the foetal/neonatal development. On the other hand, no changes in the number of spermatocytes or spermatids is no surprise in the light of the fact that Sertoli cells can only support a limited number of germ cells and the Sertoli cell number was not changed in the ERβKO males.

As previous ERβKO models expressed several splice variants that could eventually mediate some effect in ERβKO animals, Antal et al. [123], generated a new kind of ERβKO mouse devoid of any transcript downstream of exon 3 as analysed from the uterus total RNA. These oestrogen receptor β-null male mice (ERβ_ST_L−/L−) were, contrary to previously published models, sterile and lacked the ERβ protein in the prostate and testis [123]. The origin of the sterility is unknown, since the histology of the testis from young (5 months) and old (16 to 19 months) mice seems to be normal, as well as the sperm motility [123]. In a subsequent study, the authors demonstrated that ERβ_ST_L−/L− male mice had a mildly altered sexual behaviour [143]. However, the authors did not suppose that these changes were a cause of the infertility of ERβ_ST_L−/L− males, but they speculated that these males were not left to mate for a long enough period with wild-type females [143].

Interesting findings came from the last created ERβKO mice. Maneix et al. [144], used the Cre/LoxP recombination system to remove exon 3. They confirmed the deletion of exon 3 and showed that no alternative splicing of ERβ was present in the ovaries and prostate. Thus, no ERβ protein should be produced in their ERβKO mice due to two newly formed stop codons in exon 4. However, using an antibody against the C-terminal part and ligand-binding domain of ERβ protein, the protein in the ventral prostate of the wild-type and ERβKO mice was immuno-detected, while no detection was observed in the original ERβKO_CH_ mice. The authors speculate that the termination of translation in the newly formed stop codons was not efficient and that ribosomes underwent a frame shift that led to maintaining the regular reading frame of ERβ. The mutant mice expressed the ERβ protein with a disrupted DNA binding domain, while the rest of the receptor was normal. Since no defects were observed in their mutant male mice, the authors concluded that most of the physiological functions of ERβ do not involve ERE binding, and thus its action must be via a non-genomic pathway [144].

#### 2.4.4. GPER Knockout

Several studies have detected GPER in rat [94,95,98] and human [42,43,44] testicular cells. Although GPER expression has been detected in mouse germ cell-derived cell lines [46,97] and blood vessels of mouse testes [145], Otto et al. [82] did not detect any expression of GPER in the mouse testes. Therefore, it is not so surprising that GPER-deficient mice are normally fertile [82,100].

## 3. The Effect of Oestrogens on Spermatogenesis and Sperm

The physiological oestrogens’ level is essential for sperm production as their high level [146] or absence [129] leads to spermatogenesis disruption. It has been shown that oestrogens influence the testis development, thus indirectly affecting the sperm production [147]. Furthermore, it has been found that oestrogens also play a role in spermatogenesis [148], fluid reabsorption in the rete testis, and sperm migration through the male reproductive tract [1]. Initial studies were performed in vivo by manipulating the levels of oestrogens or their receptors. To study a direct oestrogen effect on the testis in vivo is not easily feasible, since changes in the oestrogen environment may also influence the levels of gonadotropins and testosterone. Therefore, it is necessary to be cautious in judging whether the observed effects are direct or indirect. Interesting information on oestrogen and oestrogen signalling importance comes from clinical studies. There are reported cases of men with infertility related to specific mutations in the genes for either oestrogen receptor [149] or P450 aromatase [150,151]. These studies describe reduced [151] or absent [150] sperm motility, as well as hypospermatogenesis and germ cell arrest mainly at the primary spermatocyte level [150]. So far, there is no evidence of GPER-related disturbed reproduction in human; however, mainly experimental studies have suggested roles of this receptor as a causative agent/modulator of physiology and disease [152].

The high intratesticular oestrogen level, induced by E2 administration, leads to changes in the expression of many genes containing ERE in their promotor sequence [146]. These deregulated genes are involved in androgen metabolism, maintenance of cytoskeletal integrity, intracellular transport and endocytosis, as well as germ cell apoptosis [146]. However, the effect of oestrogens greatly depends on the dose. High doses showed to damage spermatogenesis, while the lower ones had a positive effect. As an example, the aging of male rats is accompanied by a decrease in all the following: intratesticular testosterone and E2, expression of aromatase, ERα, ERβ genes, sperm production [153,154] and antioxidant enzymatic activities [154]. All these changes could be, at least partially, reversed by E2 administration [153,154], suggesting an additional protective role of oestrogen in the male reproduction via improvement of the cellular antioxidant defence system [154].

A recent focus on the impact of oestrogens on individual testicular cells emphasised a need for exploring various signalling pathways and characterizing a specific role of individual oestrogen receptors. Experiments were performed under in vitro conditions; hence, it was possible to study the oestrogens’ direct effect. On the other hand, cell cultures can only simulate a natural environment to a certain extent. The study of the E2 effect on mouse spermatogonial cell line GC-1 showed that a cross-talk between ERα and GPER is involved in mediating the oestrogen-activated EGFR/ERK/c-fos signalling cascade, which in turn stimulates cell proliferation [46]. E2-induced spermatogonial proliferation through ERK1/2 signalling was also proposed in non-mammalian vertebrate models [155,156]. Another effect of oestrogens was observed using an in vitro culture system for human seminiferous tubules to preserve cell association in the most natural conditions [85]. Incubation of seminiferous tubules in the serum- and hormone-free conditions led to apoptosis of mostly spermatocytes and spermatids and it was reversed by addition of the physiological concentration of E2, which had an anti-apoptotic effect on germ cells [85]. On the other hand, the in vitro effect of E2 on individual cultivated germ cells showed increased apoptosis in primary cultured rat pachytene spermatocytes [94], rat round spermatids [95] and mouse spermatocyte-derived cell line, GC-2 [97]. In particular, E2-mediated activation of ERα and GPER in spermatocytes triggered the EGFR/ERK/c-Jun signalling cascade, which consequently initiated an apoptotic pathway [94,97]. Whether ERβ also plays a certain role in E2-mediated response in spermatocytes remains a question as ERβ had not been part of the functional study. An interesting result was obtained in the case of spermatids, where ERα and GPER mediated activation of the EGFR/ERK signalling cascade, which led to promotion of apoptosis; however, the same signalling cascade activated by ERβ prevented the apoptosis [95]. This indicated that the overall E2 effects could depend on the relative representation of individual oestrogen receptors. In case of spermatids, the E2-mediated apoptotic pathway predominates [95]. The opposing results obtained for the same cells (anti-apoptotic effect) [85] (apoptotic effect) [94,95,97] may result from species specificity, selected experimental E2 concentrations, or more likely from a different approach used, where in one case [85], E2 could exert its effect also on somatic testicular cells, and thus contribute to the overall E2 effect on germ cells.

Further studies have deepened the knowledge about the direct contribution of ERs in spermatogenesis. Artificially induced over-activation of ERα or ERβ in male rats led to a decreased litter size and sperm count, as well as to an increased pre- and post-implantation loss [130]. Moreover, both ERs played a distinct role during spermatogenesis [157]. Over-activation of ERα led to the arrest of differentiation of round to elongated spermatids as a result of decreased expression of protamine 1 (*Prm1*), transition nucleoprotein 1 (*Tnp1*) and 2 (*Tnp2*), and this was accompanied with changes to the hormone level (follicle-stimulating hormone—FSH, luteinizing hormone—LH, and testosterone) [130,157]. On the other hand, over-activation of ERβ did not lead to changes in the gonadotropin level, but caused spermatocytes’ apoptosis (intrinsic pathway—via mitochondria) and spermiation failure as a result of the disruption of tubulobulbar complexes (TBCs) [130,157]. This observation is in agreement with previous studies where a high intratesticular level of oestrogens affected formation of TBCs [158] due to deregulation of actin-remodelling transcripts [102]. Apical TBCs are actin-based structures on the concave surface of the spermatid head, where a close contact between Sertoli cells and elongated spermatids is formed and spermatids are prepared for release into the tubular lumen [159]. Any disruption in TBC formation may cause failure in the spermatid detachment from Sertoli cells. As many EDs may activate ERs, this could represent one of the mechanisms on how EDs may contribute to an unfavourable trend in declining the sperm quality.

The switch of ERα/ERβ expression during postnatal development of Sertoli cells allows E2 to mediate its effects in distinct directions and regulate the proliferation of immature Sertoli cells, while later the same signal pushes these cells towards differentiation [93]. In 15-day old rats, the expression of ERα prevails and its activation triggers EGFR/MAPK3/1 and PI3K pathways that lead to the activation of NF-κB, which mediates increase in cyclin D1 expression [93,99]. On the other hand, in older males, ERβ prevails and mediates increase in the expression of genes important in differentiation and cell cycle inhibition through activation of the PI3K/CREB pathway [93]. Further, 15-day old rat Sertoli cells also express GPER [98], and its activation by E2 triggers the signalling cascades that prevent apoptosis [98,99].

In addition to the oestrogen exposure in the male reproductive system, spermatozoa are also exposed to E2 during their deposition and transport into the female genital tract. Ejaculated spermatozoa are unable to fertilise the egg in mammals. In the female genital tract, sperm have first to undergo a maturation process called capacitation [160]. In order for sperm to penetrate the egg surroundings, capacitation is followed by exocytosis of lytic enzymes during the acrosome reaction [160]. Both of these processes have been shown to be affected by oestrogens [121,122,161,162,163,164,165,166,167,168]. Since the proper timing of sperm maturation prior to reaching the egg is crucial for successful fertilization, any changes in sperm capacitation and/or acrosome reaction may lead to reduced fertility [169].

## 4. Interaction of Oestrogen-Like Compounds with ERs

As all hormonal systems, oestrogen signalling is also susceptible to disruption by an imbalance in hormone concentration. The importance of proper oestrogen signalling in males is proved by the fact that it can be disrupted by various exogenous substances with estrogenic activity, and this disruption leads to impairment of male fertility. These hormonally active substances belong to endocrine disruptors (EDs) with estrogenic activity. Some of these EDs are/were used in the pharmaceutical industry, others can be released from various products or factories and thus get to the environment or even food intake. These EDs are able to bind to ERs and directly modify their genomic and non-genomic activity, interact with transcription factors, or modify metabolic enzymes that are important for oestrogen synthesis and metabolism [170]. ERs possess relatively large ligand-binding sites. The ligand-binding site of ERα has a volume of 450 Å^3^ and that of ERβ 390 Å^3^; however E2, as the natural ligand of ERs, has a volume of only 245 Å^3^ [171,172]. This allows a diverse number of small molecules to bind to the ligand-binding site and trigger the oestrogen response.

This chapter does not aim to cover all data available on oestrogen-like compound interaction with ERs and their effect on male fertility. It should just serve as a brief overview of the effects of two EDs with estrogenic activity. They are both representatives of xenoestrogens, of either synthetic (diethylstilbestrol, DES) or natural (zearalenone, ZEA) origin. On these examples, we aim to highlight the importance and fragility of the oestrogen system in males.

One of the examples of EDs, which can bind with a very high affinity to ERs and thus influence both genomic and non-genomic ER signalling, is DES. It is a synthetic oestrogen that was used between the late 1940s and early 1970s in pharmacology to prevent abortions. However, later it was proven to be linked with an increased risk of vaginal adenocarcinoma in daughters of treated mothers and thus established as human transplacental carcinogen, which subsequently led to banning its usage [173]. Moreover, DES was later associated with reproductive tract malformations in human and female-related increased infertility or pregnancy-related issues in the descendants of the treated mothers [174]. The exposure to DES during foetal development caused permanent changes in the programming of oestrogen target tissues, which led to various abnormalities of the reproductive tract and infertility-related conditions [175]. The negative effect was observed even in men of the F2 generation whose fathers had been exposed to DES in utero [176]. The adverse effect of DES on male fertility was also proven in rats [177] and mice [178,179]. One possible mechanism was suggested in a study showing that the neonatal DES exposure induced alterations in the DNA methylation status of seminal vesicle secretory protein IV (Svs4) and lactoferrin (Ltf) genes in seminal vesicles of adult mice [180]. It was shown that alteration of these DNA methylation patterns was mediated by ERα. Further, the gene expression of three epigenetic modifiers (DNMT3A—DNA methyltransferase 3A, MBD2—methyl-CpG binding domain protein 2, HDAC2—histone deacetylase 2) was increased in DES-exposed wild-type male mice, indicating their involvement in the mediation of methylation changes in seminal vesicles [180].

Other oestrogen-like compounds are products of various plants or fungi, so called phytoestrogens. One such phytoestrogen is ZEA. It is a nonsteroidal mycotoxin with estrogenic activity, which is produced by Fusarium fungi. These fungi are common contaminants of cereal crops, and thus ZEA could become part of food and influence humans and animals. It has been shown that ZEA and its metabolites are able to compete with E2 for the specific binding sites of ERs and initiate the oestrogen-like response [181]. In vivo and in vitro studies have shown that exposure to ZEA results in decreased steroidogenesis and thus reduction of testosterone produced by Leydig cells. Moreover, the in vivo exposure of male rodents to ZEA caused various fertility-related defects such as an increased number of abnormal spermatozoa, reduced sperm count [182,183], lower pregnancy rate [182], and reduced number of spermatogonia and Sertoli cells [177]. The effect of either ZEA or E2 on ER expression in the testicular tissue and sperm was further conclusively investigated [184]. In ZEA-exposed animals, there was a reduction in ERα mRNA as well as protein in the testis and sperm, but at the same time, the expression of ERβ was upregulated in the testis or remained unchanged in the sperm. Moreover, reduced Leydig cell steroidogenesis and increased apoptosis of testicular cells was observed in the ZEA-exposed group [184]. The described effects of ZEA on the sperm quality, viability of testicular cells and steroidogenesis were very similar to those of E2-exposed rats [184], except that there were no changes in the ERβ expression compared to the ZEA-exposed group. The authors hypothesised that the mechanism of action of these two compounds is probably slightly different; nevertheless, both ZEA and E2 affected the ERα mRNA content, which in consequence probably influenced the sperm parameters [184].

All the above-mentioned data show the importance of physiological oestrogen signalling and ERs’ expression in the male reproductive tract. It is obvious that the oestrogen system plays an important role during prenatal, postnatal, as well as adult life, and its disruption leads to various defects, including alteration of sperm parameters, increased apoptosis of testicular cells, or increased susceptibility to tumour formation. Moreover, it has been shown that both of the mentioned EDs are able to influence expression of ERα and/or ERβ, and it is highly probable that the male reproductive parameters are influenced through this mechanism.

## 5. Conclusions

Despite the fact that data on ERs’ localization and/or detection are not always uniform, it is undisputable that ERα, ERβ and GPER are expressed in both testicular cells and sperm. Studies with genetically manipulated mice brought valuable knowledge about E2 and ERs’ signalling in males, confirming the important role of oestrogens and their receptors in several aspects of testicular physiology and epididymal function. Both classical ERs (ERα, ERβ) play a role in spermatogenesis, although they regulate different pathways or events, such as spermatid differentiation/spermiation and Sertoli cell differentiation/proliferation. Furthermore, the receptors for oestrogens may play synergistic (GPER and ERα signalling), as well as antagonistic (pro- and anti-apoptotic effect in spermatids) roles in the testicular cell biology, and the overall E2 effect depends on the relative expression of individual receptors. Moreover, the E2 concentration, specific cell environment and inter-cell communication are all important aspects that can modulate the E2-mediated response. Importantly, E2 signalling requires interaction with their specific receptors in order to regulate an endless list of processes occurring during spermatogenesis and sperm maturation, discussed in this review, such as proliferation of gonocytes, spermatogonia, Leydig and Sertoli cells, apoptosis of Sertoli cells, spermatocytes and spermatids, spermatid differentiation, spermiation, Sertoli cell differentiation, germ cell viability, androgen metabolism, fluid reabsorption and sperm epididymal maturation, sperm capacitation and acrosome reaction. ERs possess a binding site for natural oestrogens; however, this site can also be occupied by certain endocrine disrupting chemicals with estrogenic effects. Thus, non-physiological activation of oestrogen receptors could represent a mechanism on how EDs contribute to impaired fertility. Besides a direct interaction with ERs, many EDs are able to modulate their expression and thus affect reproductive parameters. Identification and characterization of ERs’ variants are, therefore, crucial for not only understanding the oestrogen physiology in male reproduction, but also for comprehending the mechanism of endocrine disruptors’ action.

## Abbreviations

ArKO	aromatase knockout
DES	diethylstilbestrol
E2	17β-estradiol
EDs	endocrine disruptors
ERE	oestrogen response element
ERs	oestrogen receptors
ERαKO	ER alpha knock-out
ERβKO	ER beta knock-out
ESR1/ERα	oestrogen receptor 1/alpha
ESR2/ERβ	oestrogen receptor 2/beta
GPER/GPR30	G protein-coupled oestrogen receptor 1
ZEA	Zearalenone
